# Feature Point Matching Based on Distinct Wavelength Phase Congruency and Log-Gabor Filters in Infrared and Visible Images

**DOI:** 10.3390/s19194244

**Published:** 2019-09-29

**Authors:** Xiaomin Liu, Jun-Bao Li, Jeng-Shyang Pan

**Affiliations:** 1 School of Electronics and Information Engineering, Harbin Institute of Technology, Harbin 150001, China; 17b901029@stu.hit.edu.cn; 2 Information and Electronic Technology Institute, Jiamusi University, Jiamusi 154002, China; 3 College of Computer Science and Engineering, Shandong University of Science and Technology, Qingdao 266510, China; jengshyangpan@fjut.edu.cn; 4 Fujian Provincial Key Laboratory of Big Data Minning and Applications, Fujian University of Technology, Fuzhou 350118, China; 5 College of Informatics, Chaoyang University of Science and Technology, Taichung 413, Taiwan

**Keywords:** infrared and visible image matching, phase congruency, spectral decomposition, physical wavelength, corners detection

## Abstract

Infrared and visible image matching methods have been rising in popularity with the emergence of more kinds of sensors, which provide more applications in visual navigation, precision guidance, image fusion, and medical image analysis. In such applications, image matching is utilized for location, fusion, image analysis, and so on. In this paper, an infrared and visible image matching approach, based on distinct wavelength phase congruency (DWPC) and log-Gabor filters, is proposed. Furthermore, this method is modified for non-linear image matching with different physical wavelengths. Phase congruency (PC) theory is utilized to obtain PC images with intrinsic and affluent image features for images containing complex intensity changes or noise. Then, the maximum and minimum moments of the PC images are computed to obtain the corners in the matched images. In order to obtain the descriptors, log-Gabor filters are utilized and overlapping subregions are extracted in a neighborhood of certain pixels. In order to improve the accuracy of the algorithm, the moments of PCs in the original image and a Gaussian smoothed image are combined to detect the corners. Meanwhile, it is improper that the two matched images have the same PC wavelengths, due to the images having different physical wavelengths. Thus, in the experiment, the wavelength of the PC is changed for different physical wavelengths. For realistic application, BiDimRegression method is proposed to compute the similarity between two points set in infrared and visible images. The proposed approach is evaluated on four data sets with 237 pairs of visible and infrared images, and its performance is compared with state-of-the-art approaches: the edge-oriented histogram descriptor (EHD), phase congruency edge-oriented histogram descriptor (PCEHD), and log-Gabor histogram descriptor (LGHD) algorithms. The experimental results indicate that the accuracy rate of the proposed approach is 50% higher than the traditional approaches in infrared and visible images.

## 1. Introduction

Image matching is the process of aligning images of the same scene which have been acquired under different conditions, such as with a different field of view, at different scales, different resolutions, different times, or using different sensors, and so on. It is a prerequisite step for many applications [1,2,3], such as visual navigation, precision guidance, image fusion, medical image analysis, and target location. Conte et al. proposed a vision-based navigation system which uses an image matching method to obtain useful position information from aerial imagery [4]. Zhang et al. employed the coarse-to-fine image matching methodology to achieve scene matching guidance [5]. In [6], an effective visible light and infrared image fusion algorithm, using feature residuals and statistical matching, has been proposed. Yang et al. utilized learned non-linear local descriptors and feature matching to predict pseudo-computed tomography (PCT) images from T1- and T2-weighted magnetic resonance imaging (MRI) data [7]. Recently, image matching methodologies have been widely and effectively applied in the field of the target location, presenting unique advantages [8]. For the purpose of determining the best matching relations between two images with such differences, an effective feature point detection approach is of significant use.

Infrared (IR) and visible image matching, which is utilized commonly in the fields of computer vision, target identification, and military ground vehicle identification [9], has recently attracted the attention of the research community. Huang et al. proposed an improved registration method for infrared and visible remote sensing images using NSCT and SIFT [10]. Hariharan et al. presented a new image fusion method, which utilized Empirical Mode Decomposition (EMD), for improved face recognition [11]. Cheng et al. employed infrared and visible image matching methods to complete automatic target recognition [12]. In the military field, 24 h all-weather functionality is required for the military tasks of target/threat detection and identification. In target identification, infrared image matching has been used as a supplement for visible images, in order to reduce the effects of poor illumination. Infrared and visible image matching is divided into the categories of multispectral or multiband image analysis [13]. Infrared and visible light images, captured from two sensors working in different bands, present significant distinctions from single-band methods. These distinctions include the following points: (1) different imaging mechanisms make the infrared and visible images able to deliver properties of objects in different bands. (2) Different imaging conditions cause the gray distortion and the geometric deformation of images to be easily affected by shooting time, season, environment, light intensity, and so on, which causes the infrared and visible images to differ in many characteristics. Pan et al. studied wireless sensor technology concerning deep algorithms [14,15,16]. Wang et al. proposed many approaches for heterogeneous wireless sensor networks [17,18,19]. According to the analysis of the physical wavelengths for the images, visible images possess more information than infrared.

The wavelet transform has been utilized to obtain local frequency information at a point in a signal [20]. In [21], a considerable amount of visual processing models have been shown to be quite effective in accounting for a wide range of physiological observations. Theories of cortical neuron behavior have varied widely, from Fourier analysis to edge detection. Edge detection has proven to be an effective means of describing image features. However, it has been shown that cortical neurons lack an edge detection mechanism. Thus, a new approach, based on Gabor’s theory of communication, was proposed. Gabor showed how to represent time-varying signals in terms of functions that are localized in both time and frequency. In 1985, Daugman proposed the 2D Gabor filter family, which can describe the two-dimensional receptive field profiles of simple cells in the mammalian visual cortex and is the best choice for discerning image textures [22]. Following that, Daugman extracted phase information of iris patterns to encode iris features, in order to make iris recognition more accurate [23]. Recently, the phase congruency-based Gabor filter family has been proposed for the detection of corners and edges in the images with varying contrast [24]. David showed that a feature of this model is that, on a linear scale, the bandwidths of the different sensors are proportional to their optimal bandwidths [21]. However, the distinction of images from different sensors reflects the physical wavelength difference, as was analyzed above. Similar to the physical wavelength of the light, the wavelength of the wavelet influences image feature extraction. A shorter wavelength will describe detailed image information and a longer wavelength will describe coarse image information. Thus, the wavelength should be adjusted, according to the physical wavelengths of the matched images. The wavelengths utilized for the visible range should be set to be larger, in order to extract coarse information; and that for the infrared should be smaller, in order to extract detailed information. In other words, the two images are decomposed by the wavelet, and the most similar parts are matched to obtain better matching results. Inspired by these ideas, distinct wavelength log-Gabor filters are used for feature detection of infrared and visible images in this paper.

The rest of this paper is arranged as follows. Section 2 details the related works. Then, the proposed approach is illustrated in Section 3. The experiments are presented in Section 4. Finally, the conclusions are provided in Section 5.

## 2. Related Work

A classical automatic image matching process includes the following four steps: (1) feature points detection; (2) feature points description and matching; (3) transformation model estimation; and (4) image resampling. According to the above process, most multimodal image matching methods can be broadly classified into two categories: feature-based and area-based.

Area-based methods commonly utilize a template window of a given size to detect the feature points between two images. After the template window in an image is defined, the corresponding window in the other image is found by computing the matching information according to a predefined similarity measure, such as cross-correlation [25], phase correlation [26], or mutual information [27]. The centers of the matching windows are regarded as the feature points, which are then used to align the two images.

Feature-based methods first extract the significant features from both matched images and then match them using their similarities. These significant features can be regions [28], lines [29] or points [30,31], and are regarded as features with distinctiveness and stability at fixed locations, regardless of changes of image geometry, scanned scene changes, non-linear intensity changes, and so on. Recently, local invariant features have been widely applied in image matching, such as SIFT [32], SURF [33], BRISKLeu [34], ORB [35], WLD [36], WLBP [37] and so on, which have been broadly used in applications for visible image matching due to their robustness to geometric and illumination changes [38]. However, inferior matching performance has been reported for these methods when used with multispectral images.

A host of researchers have made significant efforts towards matching infrared and optical images. In the 1990s, a visible–infrared matching method using a multi-scale edge detection algorithm to obtain surface boundaries was first presented, in which the matching system used a hierarchical estimation process [39]. Following that, many variants of SIFT have been advocated for visible–infrared image matching. Firmenich et al. recommended the gradient-invariant SIFT (GDSIFT) as an RGB–NIR image matching method [40]. The gradient orientation modification SIFT (GOM-SIFT) method has been employed by Yi et al [41]. On the other hand, the gradient information differs between the visible and infrared images, which can cause these variants to perform worse than the SIFT method [42]. The discrete curve evolution (DCE) method has also been advocated, which gives better results for planar scenes. However, it cannot be well-adapted to non-planar scenes [43].

Recently, the local self-similarity frequency descriptor has been put forward [44], which allows for robust descriptors in multispectral image matching [45]. More studies have detailed the advances in visible and infrared image matching [46,47]. Concerning the methods which deal with the special application of handling multispectral images, the descriptor edge-oriented histogram (EOH) has been considered to be a dramatic baseline in scientific studies [46,47,48,49]. However, it has been shown that it is difficult to select an appropriate threshold for the EOH descriptor, in order to ensure that the edges extracted from multispectral images are similar [48]. Thus, Fu et al. advocated a local feature descriptor with a combination of structural and textural information for multispectral image matching, which is perfect for the non-linear intensity changes of multispectral images [50].

In the early 1970s, a number of attempts were made to reveal the purpose of the rather mysterious behavior of cortical neurons. It is evident that cortical neurons are selective to spatial frequency and orientation, which directed a number of researchers to produce something like a Fourier transform. In [21], two types of feature descriptions—Gabor and log-Gabor—have been considered as good methods for transforming redundancy. Gabor filtering is similar to the operations of simple cells in the visual cortex, which was presented by Daugman, in 1980, as a framework for understanding the orientation- and spatial frequency-selective receptive field properties of neurons in the brain’s visual cortex, which outperformed the classical dyadic wavelet transform for texture classifications in many tasks [51]. Recently, log-Gabor filters have been advocated for replacing EOH filters, as they have shown better performance than other methods [46,52]. In these approaches, fast feature detection is utilized to detect the feature point [53], which has no adaptability for the descriptor, based on the log-Gabor filters. However, feature detection is an important step in the image matching process. To address this problem, a corner and edge detector have been developed from the phase congruency model of feature detection, which provides information invariant to image contrast [24]. Recently, many researchers have used phase congruency methods for feature detection [54,55,56,57].

## 3. Feature Point Matching Based on Distinct Wavelength Phase Congruency and Log-Gabor Filters for Infrared and Visible Images

Inspired by the theory of phase congruency and Gabor filters, we present a feature point matching method based on distinct wavelength phase congruency and log-Gabor filters for infrared and visible images.

**Figure 1:**
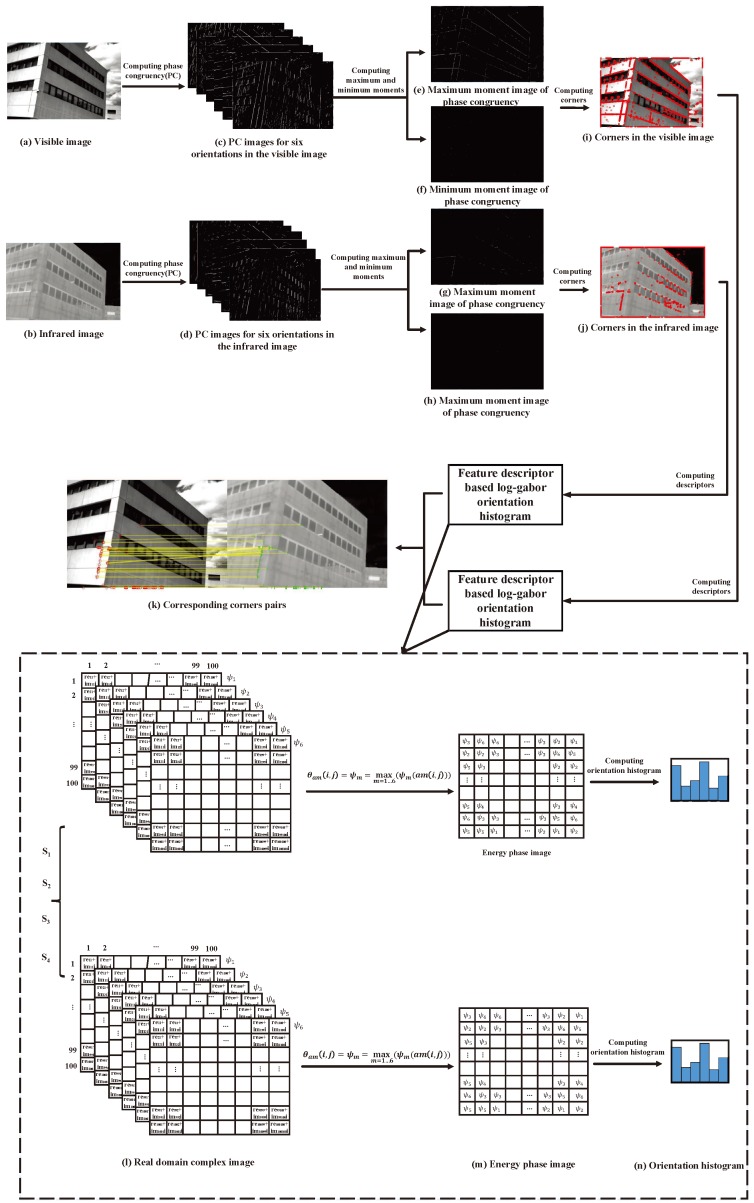
Overview of feature point matching based on phase congruency and log-Gabor filters for infrared and visible images: (**a**,**b**) show the target image and the reference image; (**c**,**d**) show phase congruency (PC) images for six orientations in the visible and infrared images; (**e**–**h**) show maximum and minimum moments of the phase congruency for the visible and infrared images; (**i**,**j**) show the corners in the visible and infrared images; (**k**) shows the corresponding pairs; (**l**) shows the complex images in the real domain obtained with log-Gabor filters; (**m**) shows the phase image with maximum energy; and (**n**) shows the orientation histograms of the phase images.

Figure 1 gives an overview of the proposed approach. First, the original target and reference images are input, as shown as Figure 1a,b, which are dealt with, using phase congruency (PC) theory, to obtain the PC images, as shown in Figure 1c,d. Then, the maximum and minimum moments of the PC images, as shown as Figure 1e–h, are computed to obtain the corners in the images, as shown in Figure 1i,j. Next, the complex images in the real domain, as shown in Figure 1l, are computed by the log-Gabor filter, using four scales and six orientations. The phase information with maximum energy is then utilized to form the phase images at each scale, as shown in Figure 1m, and the orientation histograms of the phase images are utilized to describe the image features, as shown in Figure 1n. Finally, the nearest neighbor and RANSAC algorithms are used to determine the corresponding keypoints, as shown in Figure 1k.

### 3.1. Corner Detection Based on Distinct Wavelength Phase Congruency

Keypoints are defined as a class of important image features with large changes in intensity, such as line endings, corners, junctions, and so on, where the Fourier components of the image are typically maximum in phase. The quality of the keypoints has a direct impact on image matching. Phase congruency is a dimensionless quantity which finds keypoints in images with multispectral changes, which presents significant advantages over gradient-based methods. The local energy model of feature detection suggests that keypoints are determined at points of maximum phase congruency in an image. In images obtained from different sensors, there will be similar spectral qualities in two matched images. Thus, in our proposed method, the wavelength of the wavelet is adjusted according to the physical wavelength ranges of images from different sensors.

**Figure 2:**
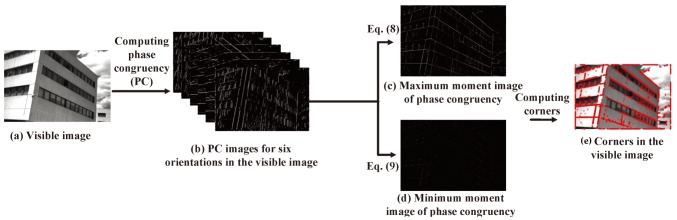
Illustration of corner detection.

Morrone and Owens defined the phase congruency function, in terms of the Fourier series expansion of a signal at a point, as [58]:

(1) $$\begin{array}{c} PC\left(x\right)=max_{\bar{\phi }\left(x\right)\in \left[0,2\pi \right]}\frac{\sum_{n}A_{n}cos\left(\phi_{n}\left(x\right)-\bar{\phi }\left(x\right)\right)}{\sum_{n}An}, \end{array}$$

where $A_{n}$ represents the amplitude of the *n*th Fourier component and $\phi_{n}\left(x\right)$ represents the local phase of the Fourier component at location *x*. The value of $\bar{\phi }\left(x\right)$ which maximizes this equation is the amplitude weighted mean local phase angle of all Fourier coefficients at the considered point. For the sake of obtaining better localization without sensitivity to noise, a new measure [24] is defined as

(2) $$\begin{array}{c} PC_{n}\left(x\right)=\frac{\sum_{n}W\left(x\right)\left\lfloor A_{n}\left(x\right)\left(cos\left(\phi_{n}\left(x\right)-\bar{\phi }\left(x\right)\right)-\left|sin(\phi_{n}\left(x\right)-\bar{\phi }\left(x\right))\right|\right)-T\right\rfloor }{\sum_{n}A_{n}\left(x\right)+\epsilon }, \end{array}$$

where W(x) is a weighting factor for frequency spread and ϵ is used to avoid division by zero. Only energy values exceeding *T* are counted in the result. As shown in Figure 2, given an input image (Figure 2a), PC images for six orientations (Figure 2b) are computed using Equation (2). Then, the covariance matrix is computed, and its minimum and maximum moments are calculated in order to produce a highly localized operator, which is then used to identify both edges and corners in a contrast-invariant manner. The covariance data is built as follows:

(3) $$\begin{array}{c} Covx(\theta )=PC(\theta )cos(\theta ), \end{array}$$

(4) $$\begin{array}{c} Covy(\theta )=PC(\theta )sin(\theta ), \end{array}$$

(5) $$\begin{array}{c} a=\sum Covx{\left(\theta \right)}^{2}, \end{array}$$

(6) $$\begin{array}{c} b=2\sum Covx(\theta )Covy(\theta ), \end{array}$$

(7) $$\begin{array}{c} c=\sum Covy{\left(\theta \right)}^{2}, \end{array}$$

where PC(θ) is the phase congruency value determined at orientation θ. The maximum and minimum moments—*M* and *m*, respectively—are computed as

(8) $$\begin{array}{c} M=\frac{1}{2}\left(c+a+\sqrt{b^{2}+{\left(a-c\right)}^{2}}\right), \end{array}$$

(9) $$\begin{array}{c} m=\frac{1}{2}\left(c+a-\sqrt{b^{2}+{\left(a-c\right)}^{2}}\right). \end{array}$$

Figure 2c,d show the maximum and minimum moment images, respectively. When *M* is greater than the threshold TM, an edge will be marked; when m is greater than the threshold Tm, a corner of an edge in the image will be detected, as shown in Figure 2e.

**Figure 3:**
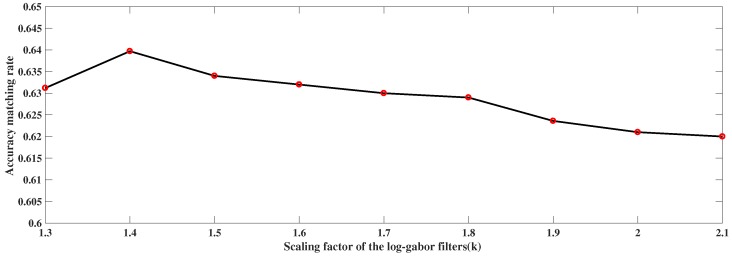
Trend of matching accuracy rate at different values of *k*.

In our work, log-Gabor filters are used to obtain frequency information local to a point in an image. The log-Gabor transform in the frequency domain, using polar co-ordinates, is given as follows:

(10) $$\begin{array}{c} G\left(r,\theta ,\psi ,n\right)=exp\left(-\frac{{\left[log\left(\frac{r}{\lambda k^{n}}\right)\right]}^{2}}{2\sigma^{2}}\right)exp\left(-\frac{{\left(\theta -\psi \right)}^{2}}{2}\right), \end{array}$$

where *r* and θ denote the radius and filter angle (in polar coordinates), *n* represents the orientation angle, σ represents the Gaussian standard deviation of the filter, λ represents the smallest wavelength of the filter, and *k* denotes the scaling factor between successive filters, which controls the wavelength of the log-Gabor filter. Images smoothed by the log-Gabor filter at different wavelengths can be considered to be images including special spectral information. Thus, in the experiment, the wavelengths of the log-Gabor filter for the matched images are adjusted respectively, in order to obtain similar spectral information from the visible and infrared images. For the visible image matching, it has better performance, in traditional approaches, when the value of *k* is set to 2.1. As shown in Figure 3, the matching accuracy rate will change when *k* is adjusted for the infrared images while k=2.1 for the visible images. Figure 3 shows that *k* = 1.4 gave the highest matching accuracy rate. Thus, given the physical wavelength range of the two matching images, we can determine certain wavelet wavelengths for the images from different sensors. Thus, the proposed method can be generalized to images from heterologous sensors.

### 3.2. Corners Detection Combining Distinct Wavelength Phase Congruency of Original Images and Gaussian Smoothing Images

It is not enough to only use the phase congruency of the original images. In [59], a corner detection algorithm based on Gaussian smoothing has been proposed and an improved corner detection algorithm based on Gaussian smoothing has been put forward by Wang et al. [60]. The proposed approaches combined the phase congruency of the original images and the Gaussian smoothed images to obtain more accurate feature points, in which maximum and minimum moments of the phase congruency for the Gaussian smoothed images were considered as the weighted image added to the corresponding moments of the original image. A two-dimensional Gaussian function is used as a tool for analysis in the linear scale-space and is defined as

(11) $$\begin{array}{c} G\left(x,y,\sigma \right)=\frac{1}{2\pi \sigma^{2}}e^{-\frac{x^{2}+y^{2}}{2\sigma^{2}}}. \end{array}$$

**Figure 4:**
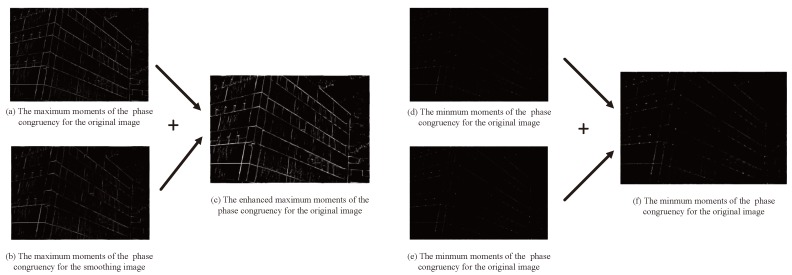
Enhanced moments of the phase congruency images.

In Figure 4a is the maximum moment of the phase congruency for the original image and Figure 4b is the maximum moment of the phase congruency for the smoothed image; Figure 4c is the enhanced maximum moment of the phase congruency obtained by adding Figure 4a,b; Meanwhile, Figure 4d,e are the minimum moment of the phase congruency for the original and smoothed images, respectively; And Figure 4f is the enhanced minimum moment of the phase congruency. Finally, the corners are detected by the enhanced maximum and minimum moment images.

### 3.3. Feature Descriptor Based on Log-Gabor Filters and the Corresponding Keypoint Detection Using the RANSAC Algorithm

**Figure 5:**
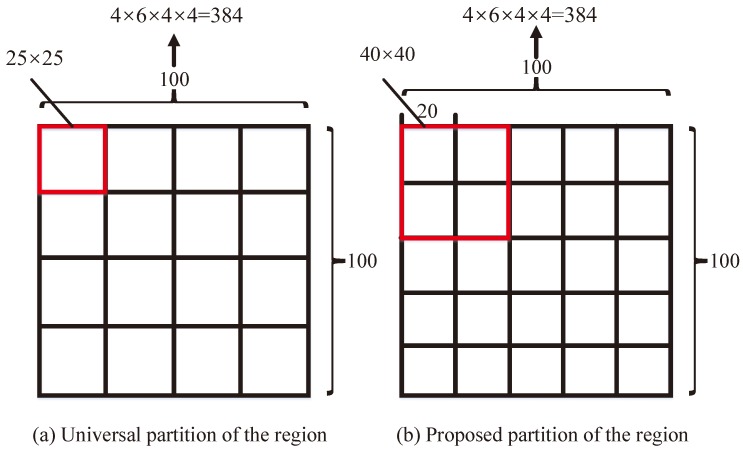
Partition of the neighborhood of a certain pixel.

A Gabor wavelet is a series of Gaussian envelopes of plane waves, which can extract spatial frequencies and local structural characteristics within the local area of the images in multiple directions and has some tolerance to changes in the spectrum, displacement, deformation, rotation, scaling, and illumination. In this paper, log-Gabor filters are proposed to describe the image features which exceed that of Gabor filters, due to the independence of the DC component [61]. As shown in Figure 1l, we obtain 24 energy images corresponding to four scales and six orientations and, then, the orientation information with maximum energy for a pixel is determined, in order to obtain the orientation image (Figure 1m). Finally, the orientation histogram is calculated (with six bins) for the orientation image, which is regarded as representing the feature descriptors, as shown in Figure 1n. For the four scales, we obtain a 4×6 dimensional vector, which can be regarded as the feature descriptor. As shown in Figure 5, we usually divide the 100×100 region centered on a given pixel into 16 subregions of size 25×25, from which we can obtain 16 histograms with 4×6 bins (i.e., giving a 4×6×4×4 dimensional vector) as the feature descriptors. However, in our approach, we selected a 40×40 subregion with an interval of 20 pixels for rows and columns in a given 100×100 region, which allows us to obtain the same 16 subregions and obtain a 4×6×4×4 dimensional vector as a feature descriptor for a given pixel. The experiment illustrated that large, overlapping subregions include more information, increasing the performance of image matching.

**Figure 6:**
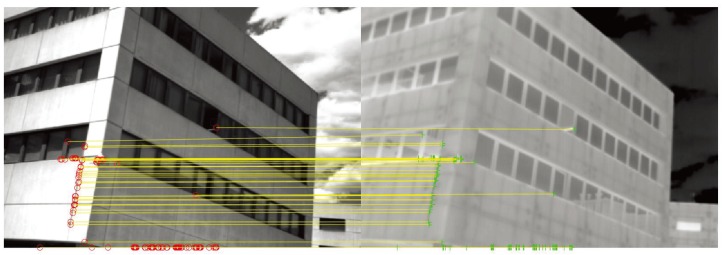
Illustration of corresponding feature point matching in the visible and infrared images.

In our experiment, the nearest neighbor distance ratio matching similarity metric was used to find corresponding keypoints. Two keypoints from the reference image and object image were considered coincident when they satisfied the following equation:

(12) $$\begin{array}{c} D\left(d^{i},d^{j}\right)<thD\left(d^{i},d^{k}\right), \end{array}$$

where D(.,.) is the Euclidean distance, $d^{i}$ is the keypoint in the reference image, $d^{j}$ and $d^{k}$ are the first and the second closest keypoints to $d^{i}$ in the target image, respectively, and th is the threshold of the distance, which was obtained based on experience. Finally, the RANSAC algorithm was used to reduce the outliers; the result of the corresponding keypoint pairs is shown in Figure 6. The experimental results showed that the proposed partition approach had a perfect effect.

### 3.4. Similarity Computation of the Points Sets from the Visible and Infrared Images by BiDimRegressional Regression Modeling

For realistic application, we need to know about the similarity between two points sets from infrared and visible images to determine whether the image matching is successful or not. Thus, BiDimRegressional regression modeling was proposed to compute the similarity for geometrics of two points sets in the infrared and visible images. The main purpose of the bidimensional regression was to estimate the degree of correspondence between two plane patterns of point locations as the points sets shown in the infrared and visible images [62]. In our experiments, BiDimRegression package has been implemented and tested in ordinary R, whose input parameter is coord containing the coordinates of the independent (A,B) and the dependent configurations (X,Y).

1. Vectors A,B represent the coordinates of the visible image, which are extracted from the independent image possessing the relationships with the corresponding infrared image from the same scene. A and B are known as the first and the second dimension, respectively.
2. Vectors X,Y represent the coordinates of the infrared image, which are extracted from the independent image possessing the relationships with the corresponding visible image from the same scene. X and Y are known as the first and the second dimension, respectively.

In this algorithm, the main output values include: $R^{2}$, *F*, *p*.

1. $R^{2}$ is the squared regression coefficient.
2. *F* represents *F* statistics for the overall regression model including appendant degrees of freedom(df1,df2).
3. *p* value is the accordant significance level.

By comparing the coordinates of the points sets, we found a very high correlation for the images with higher AR (e.g., for the Euclidean $r=0.999,F=7253.802,p=2\times 10^{-16}$) and a very low correlation for the images with lower AR (e.g., for the Euclidean r=0.651,F=2.987,p=0.16). Thus, we can determine the similarity of the infrared and visible images by the range of the parameters in realistic applications.

## 4. Experiments

In this section, we first describe the experimental data and evaluation measures. Then, four algorithms are compared and analyzed. The performance of the proposed approach is illustrated on visible and infrared images. The experiments show encouraging results.

### 4.1. Experiment Data

In order to demonstrate the advantages of the proposed approach for visible and infrared image matching, it was tested on four different data sets which have been widely used in the literature. The data sets used in the experiments are freely available, which guarantees the reproducibility of the results, and can also be utilized to improve other approaches. The Potsdam, NIR, and RGB-LWIR data sets can be found in [46] and the Multimodel Stereo Data set 2 can be obtained in [63].

**Figure 7:**
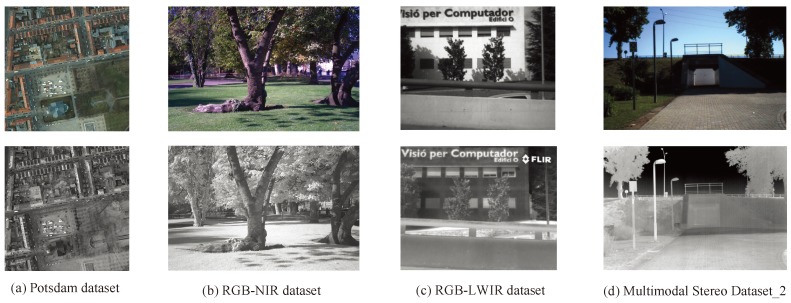
Examples from the data set.

As shown in Figure 7a, the first data set is the Potsdam data set, which is a data set of remote sensing images distributed by the International Society for Photogrammetry and Remote Sensing. It includes 38 aerial images extracted from a larger TOP (true orthophoto) mosaic, in the form of TIFF with different channel compositions. In each channel, it has a spectral resolution of 8 bits and all images have dimension 6000×6000 pixels, including the IRRG, RGB, and RGBIR forms. In our experiment, the RGBIR forms were used to measure the proposed algorithms.

The second data set is the RGB-near infrared (NIR) dataset, shown in Figure 7b, which consists of 477 images in nine categories obtained in RGB and near-infrared. The images were captured with separate exposures from modified SLR cameras, using visible and NIR filters. The scenes include country, field, forest indoor, mountain, old buildings, street, urban, and water, and all images have dimension 1024×768 pixels. The country data set was utilized in the algorithm comparison experiment.

The third data set is the RGB-LWIR data set, as shown in Figure 7c, which was proposed in a previous study. It consists of 44 pairs of visible spectrum images and long-wave infrared images (LWIR), where all images are of size 639×431 pixels. The LWIR band is the most distant infrared band from the visible spectrum, and the image pairs mostly include common shape information, while most of the texture information is missing.

The fourth data set is the Multimodal Stereo Data set 2, which was used to supplement the evaluation of the proposed algorithm, as shown in Figure 7d. This data set contains outdoor images of different urban scenes, consisting of 100 pairs of VS-LWIR images of different outdoor urban scenarios. All images have size 506×408 pixels. All the images were rectified and aligned, so that matches could be obtained in horizontal lines. Using this data set, it was further proved that the proposed algorithm was superior to the state-of-the-art algorithms.

In these data set, the matching precision for the first two data set was higher than that in the other two data sets. This fact was principally due to the spectral band closeness of the image pairs. The NIR spectrum is the closest infrared band to the visible spectrum; however, the LWIR band is the most distant infrared band from the visible spectrum.

### 4.2. Evaluation Measures

**Figure 8:**
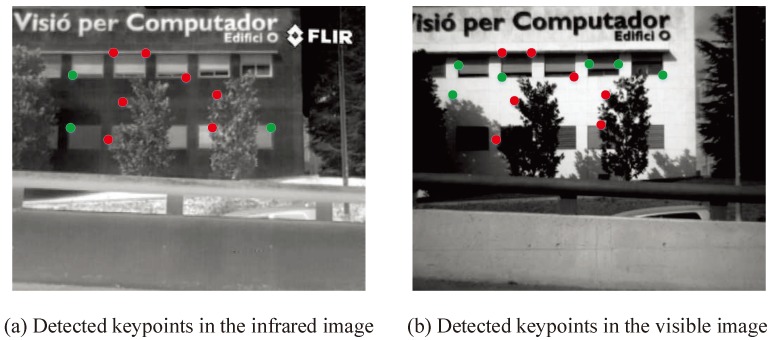
Illustration of the repeatable keypoints.

**Figure 9:**
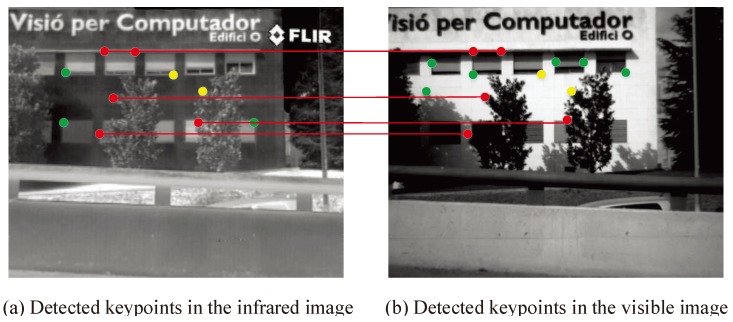
Illustration of the recall rate (RR) for the keypoints.

**Figure 10:**
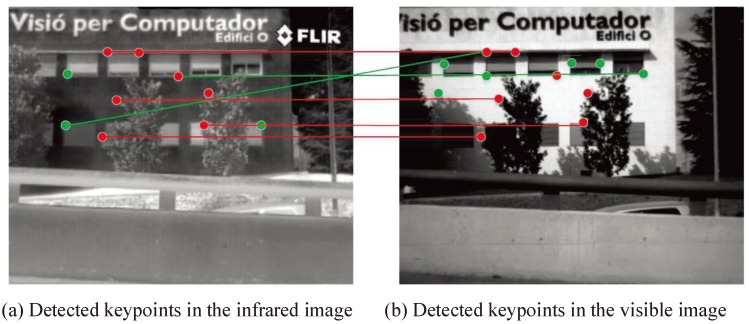
Illustration of the accuracy rate (AR) for the keypoints.

**Figure 11:**
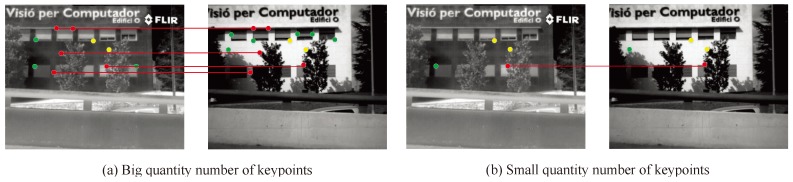
Illustration of the quantity rate (QR) for the keypoints.

In some sense, an ideal local feature should be a point defined geometrically with a location in space, but no spatial extent. In practice, images are discrete with a smallest spatial unit, known as a pixel, whose discrete property plays an important role. Ideally, one needs such local features with semantically meaningful object parts. However, this is unfeasible in practice, since this would demand a a deep interpretation of the scene content, which is not available at an early stage. On the contrary, local features can be employed to represent intensity patterns directly. At present, many evaluation measures have been proposed for the evaluation of the performance of image matching [64,65,66]. However, these evaluation measures are typically incomplete. Good features usually possess six properties [67]: repeatability, distinctiveness/informativeness, locality, quantity, accuracy, and efficiency. For measuring detector performance, according to these six properties, we advocate the following five evaluation measures:

1. Repeatable rate (RPR): as shown in Figure 8, the red points show repeatable keypoints and the green points show non-matching keypoints. In two images (the infrared image in Figure 8a and the visible image in Figure 8b), the high percentage of repeatable keypoints in both images is called repeatability. Thus, the repeatable rate (RPR) is defined as follows:
  (13) $$\begin{array}{c} RPR=\frac{CKN}{KN}, \end{array}$$
  where CKN and KN are the numbers of true corresponding keypoints and total keypoints, respectively. A higher RPR value denotes that the keypoint detection approach has a better performance.
2. Recall rate (RR): this is shown as Figure 9, he red keypoints are the true matched corresponding keypoints and the yellow ones are non-matched keypoints. A better keypoint detection algorithm should detect more corresponding keypoints accurately over the repeatable keypoints set. Thus, the recall rate (RR) can be defined as:
  (14) $$\begin{array}{c} RR=\frac{DTMPN}{DTMPN+UDTMPN}, \end{array}$$
  where DTMPN and UDTMPN are the detected true matched point number and the undetected true matched point number, respectively. The higher the value of the RR is, the better the performance of the matching approach.
3. Accuracy rate (AR): as shown in Figure 10, the detected corresponding keypoints should be accurate; the red lines show accurately detected corresponding keypoints and the green lines represent those which were not accurate. More red lines denote better performance of the image matching. Thus, the accuracy rate (AR) is defined as:
  (15) $$\begin{array}{c} AR=\frac{DTMPN}{DCPN}, \end{array}$$
  where DTMPN and DCPN are the numbers of detected true matched points and detected corresponding points, respectively. The higher the AR is, the better the performance of the matching approach.
4. Quantity rate (QR): Figure 11a demonstrates that a larger number of keypoints will generate more corresponding keypoints favorable to image matching. Figure 11b, in contrast, shows that a smaller number of keypoints can cause failure in image matching. Thus, the number of detected keypoints should be sufficiently large; for example, a reasonable number of keypoints should be detected, even on small objects. Nevertheless, the optimal number of features depends on the application. Thus, we define the QR as:
  (16) $$\begin{array}{c} QR=\frac{DKN}{IPN}, \end{array}$$
  where DKN and IPN are the numbers of detected keypoints and image pixels, respectively. A higher QR leads to better performance of the image matching, but also results in a slower speed of image matching. Thus, an adaptive QR should be determined for special applications.
5. Efficiency (EF): The detection of features in an image should be considered as time-critical application. Thus, we defined the EF as:
  (17) $$\begin{array}{c} EF=T_{CKD}+T_{CKDM}+T_{OR}, \end{array}$$
  where $T_{CKD}$, $T_{CKDM}$, and $T_{RO}$ are known as the times of candidate keypoint detection, corresponding keypoint detection, and outlier removal, respectively. In order to satisfy time-critical applications, we must make EF small enough.

### 4.3. Experiment Results Comparison and Discussion

The proposed algorithm was evaluated on the four data sets using the five evaluation measures and was compared with five existing algorithms, where the results showed that the proposed algorithm had better matching performance. The compared algorithms were the edge-oriented histogram descriptor (EHD) [68], phase congruency edge-oriented histogram descriptor (PCEHD) [9], and log-Gabor histogram descriptor (LGHD) [46] algorithms.

1. Edge-oriented histogram descriptor (EHD): this algorithm first detects the contour of the image and, then, the edge histogram descriptor is obtained by using the MPEG-7 standard [69].
2. Phase congruency edge-oriented histogram descriptor (PCEHD): This algorithm uses phase congruency to detect corners and edges in the image. In the corners, the EHD algorithm is utilized to obtain the feature descriptors.
3. Log-Gabor feature descriptor (LGHD): this algorithm uses a fast algorithm to detect corners and log-Gabor filters to generate feature descriptors.
4. Phase congruency log-Gabor image matching (PCLGM): This algorithm uses phase congruency to detect corners and edges in the image. In these corners, log-Gabor histograms are utilized to obtain the feature descriptors in overlapping subregions.
5. Modified phase congruency log-Gabor image matching(MPCLGM): This algorithm modified corners detection based on phase congruency by combining the original images and Gaussian smoothed images. In these corners, log-Gabor feature descriptors are utilized to obtain the feature descriptors.
6. Distinct modified phase congruency log-Gabor image matching(DMPCLGM): Distinct wavelengths are employed in the DMPCLGM for the visible and infrared images.

**Figure 12:**
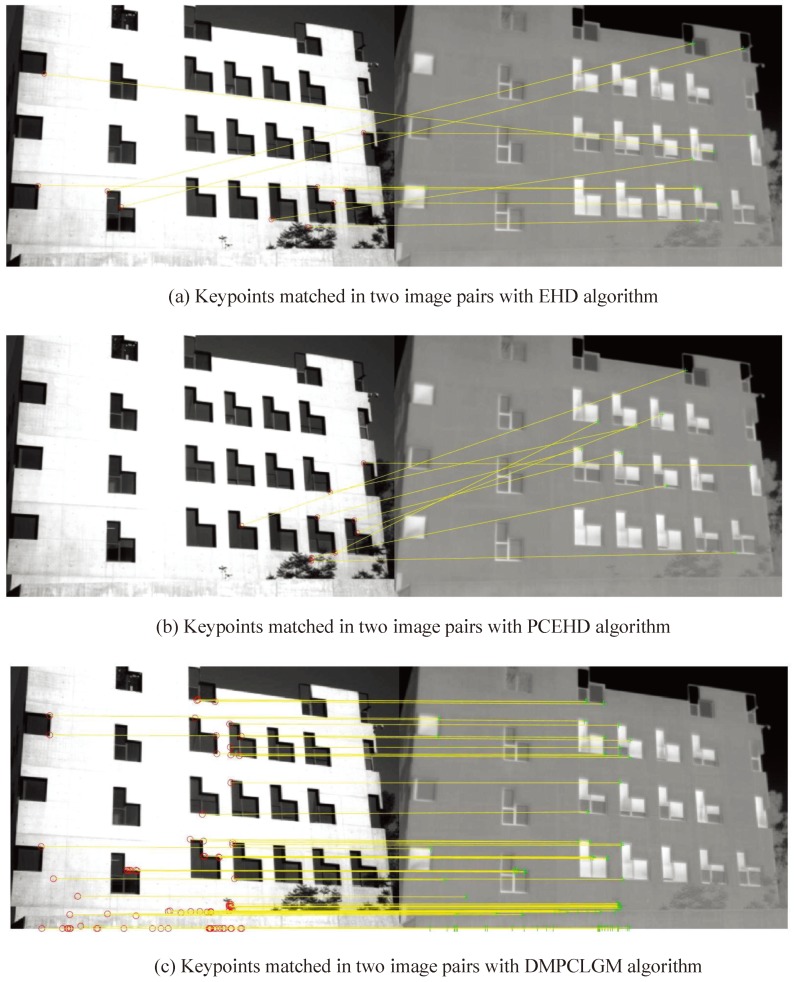
Keypoints matched in two image pairs.

Figure 12 demonstrates the results obtained with one pair of VS-LWIR images using EHD, PCEHD, LGHD, and the proposed approach. It can be observed that the AR of EHD and PCEHD were 0.015 and 0.01, respectively, and the AR of the proposed approach was 91.5%. This result shows that the proposed approach must have advantages over the other approaches. Table 1, Table 2, Table 3 and Table 4 present the comparison results for the RGB-LWIR, Multimodal Stereo 2, RGB-NIR, and Potsdam data sets respectively.

In Table 1, it can be seen that PCLGM, MPCLGM, and DMPCLGM had higher RPR, AR, and QR, but the RR was lower. In image matching methods, AR is more necessary than RR, which means that higher AR was more meaningful. The results of the comparisons for the six algorithms illustrate that combining the phase congruency of the original images and the smoothed images to detect corners and extracting the feature histogram through subregion extraction played an important role in image matching using different wavelengths of the wavelet for the visible and infrared images. For DMPCLGM, the wavelengths of the wavelets for the visible and infrared images were set to 2.1 and 1.4, which advanced the performance of the algorithm.

In Table 2, the results were similar to those for the RGB-LWIR data set, which presented higher RPR, AR, and QR, while the RR was lower. It was, thus, also shown that the proposed methods presented advantages over the state-of-the-art algorithms when used for visible and infrared images.

In Table 3 and Table 4, for DMPCLGM, all wavelengths of the wavelet for the visible and infrared images were set to 2.1, due to the similarities of the physical wavelengths. The compared results show that all the approaches had higher AR. However, the proposed algorithm showed no special advantages. Therefore, our proposed algorithm still needs to be improved to suit images with different physical wavelengths.

As shown in Table 1, Table 2, Table 3 and Table 4, the proposed algorithm demonstrated perfect performance. The lower QR at the corners caused the state-of-the-art methods to present lower performance. However, the higher QR at the corners also caused lower speeds. Thus, it is necessary to guarantee a balance between QR and AR in the proposed algorithm.

## 5. Conclusions

This paper presented a feature point matching approach based on distinct wavelength phase congruency and log-Gabor filters for infrared and visible images. First, the use of phase congruency was proposed to determine corners in the images. Generally, it is not proper to set the same wavelengths for PC when using two matched images with different physical wavelengths. Thus, the PC wavelengths for the two images were determined experimentally. Next, the moments of the PCs for the original image and Gaussian smoothed image were combined to detect the corners. Finally, log-Gabor filters were used and overlapping subregions were extracted for generating the descriptors. Five evaluation measures were employed for testing the performance of the algorithm. The experimental results show that the proposed algorithm with different wavelengths for infrared and visible images showed superior performance than other state-of-the-art approaches. Combining the original image and Gaussian smoothed image for computing the moments of the PCs also advances the performance of the algorithm. The BiDimRegression regression modeling is proposed to determine the similarity of the infrared and visible images in realistic applications.

Image matching for visible and infrared images presents many challenges for images captured using different sensors, which requires more effort to increase the performance of the approaches. The proposed approaches still have two main problems: (1) although the proposed approach shows some advantages over other methodologies, it still has a lower accuracy rate, and (2) due to the complexity of the algorithm, its runtime is too long. In the future, we will study how to remove the outlier points in order to increase the accuracy rate and adjust the number of keypoints to reduce the runtime. Meanwhile, we will extend the proposed approach to multimodal image matching.

## Figures and Tables

**Table 1:** Comparison of the six algorithms using the RGB-LWIR data set.

Descriptor	RPR	RR	AR	QR	EF
EHD	0.02	0.04	0.14	0.005	0.28
PCEHD	0.02	0.02	0.01	0.005	1.05
LGHD	0.02	0.02	0.01	0.005	1.55
PCLGM	0.19	0.014	0.59	0.27	40.3
MPCLGM	0.198	0.013	0.62	0.31	65.54
DMPCLGM	0.27	0.01	0.64	0.46	82

**Table 2:** Comparison of the six algorithms using the Multimodal Stereo Data set 2.

Descriptor	RPR	RR	AR	QR	EF
EHD	0.025	0.054	0.1	0.0018	0.4
PCEHD	0.034	0.037	0.123	0.0025	1.87
LGHD	0.04	0.08	0.25	0.003	11.56
PCLGM	0.24	0.003	0.21	0.43	67.68
MPCLGM	0.24	0.013	0.26	0.43	71.62
DMPCLGM	0.24	0.0075	0.32	0.43	78.01

**Table 3:** Comparison of the six algorithms using the RGB-NIR data set.

Descriptor	RPR	RR	AR	QR	EF
EHD	0.087	0.3	0.91	0.11	8.65
PCEHD	0.087	0.3	0.9	0.11	11.41
LGHD	0.087	0.4	0.93	0.11	30.3
PCLGM	0.2976	0.0282	0.8991	0.2279	30.51
MPCLGM	0.2976	0.0278	0.8919	0.22341	30.40
DMPCLGM	-	-	-	-	-

**Table 4:** Comparison of the six algorithms using the Potsdam data set.

Descriptor	RPR	RR	AR	QR	EF
EHD	0.22	0.27	0.99	0.06	4.48
PCEHD	0.22	0.27	0.99	0.06	5.73
LGHD	0.22	0.28	1	0.06	15.16
PCLGM	0.22639	0.0528	0.7234	0.3134	69.05
MPCLGM	0.22639	0.0604	0.7261	0.4170	96.1237
DMPCLGM	-	-	-	-	-
